# Plastome Sequences Help to Resolve Deep-Level Relationships of *Populus* in the Family Salicaceae

**DOI:** 10.3389/fpls.2019.00005

**Published:** 2019-01-22

**Authors:** Dan Zong, Peihua Gan, Anpei Zhou, Yao Zhang, Xinlian Zou, Anan Duan, Yu Song, Chengzhong He

**Affiliations:** ^1^Key Laboratory for Forest Genetic and Tree Improvement and Propagation in Universities of Yunnan Province, Southwest Forestry University, Kunming, China; ^2^Key Laboratory of Biodiversity Conservation in Southwest China, State Forestry Administration, Southwest Forestry University, Kunming, China; ^3^Key Laboratory for Forest Resources Conservation and Utilization in the Southwest Mountains of China, Ministry of Education, Southwest Forestry University, Kunming, China; ^4^Center for Integrative Conservation, Xishuangbanna Tropical Botanical Garden, Chinese Academy of Sciences, Mengla, China; ^5^Southeast Asia Biodiversity Research Institute, Chinese Academy of Sciences, Nay Pyi Taw, Myanmar

**Keywords:** *Populus*, Salicaceae, chloroplast genome, phylogenetic, morphological traits

## Abstract

*Populus*, a core genus of Salicaceae, plays a significant ecological role as a source of pioneer species in boreal forests. However, interspecific hybridization and high levels of morphological variation among poplars have resulted in great difficulty in classifying species for systematic and comparative evolutionary studies. Here, we present phylogenetic analyses of 24 newly sequenced *Populus* plastomes and 36 plastomes from GenBank, which represent seven genera of Salicaceae, in combination with a matrix of eighteen morphological characters of 40 *Populus* taxa to reconstruct highly supported relationships of genus *Populus*. Relationships among the 60 taxa of Salicaceae strongly supported two monophyletic genera: *Populus* and *Salix*. *Chosenia* was nested within the genus *Salix*, and five clades within *Populus* were divided. Clade I included the three taxa *P. euphratica, P. pruinosa*, and *P. ilicifolia*. Clade II contained thirteen taxa [*P. adenopoda, P. alba, P. bolleana, P. davidiana, P. hopeiensis, P. nigra, P. qiongdaoensis, P. rotundifolia, P. rotundifolia* var. *duclouxiana, P. tremula, P. tremula × alba, P. tomentosa*, and *P. tomentosa* (NC)]. Clade III included the ten taxa *P. haoana, P. kangdingensis, P. lasiocarpa, P. pseudoglauca, P. qamdoensis, P. schneideri, P. simonii, P. szechuanica, P. szechuanica* var. *tibetica*, and *P. yunnanensis*. Clade IV included *P. cathayana, P. gonggaensis, P. koreana, P. laurifolia, P. trinervis, P. wilsonii*, and *P. xiangchengensis*. The last clade comprised *P. angustifolia, P. balsamifera, P. deltoides, P. deltoides × nigra, P. fremontii, P. mexicana*, and *P. trichocarpa*. This phylogeny is also supported by morphological traits, including bark smoothness, bud size, petiole shape, leaf inflorescence, male anther length and male anther tip.

## Introduction

The family Salicaceae is primarily distributed in cold, tropical and warm temperate regions worldwide and is a dioecious woody and shrub plant throughout the northern hemisphere (Leskinen and Alström-Rapaport, 1999; Wang et al., 2014). Salicaceae includes over 50 genera, which comprise nearly 1000 species (Stevens, 2001; Chase et al., 2002; Byng et al., 2016). Phylogenetic analyses of Salicaceae using DNA sequences from the chloroplast gene *rbcL* (Azuma et al., 2000; Chase et al., 2002) and *ITS* of nuclear rDNA (Leskinen and Alström-Rapaport, 1999) strongly suggest that *Populus* and *Salix* are both monophyletic.

The species of the genus *Populu*s, commonly known as poplars, aspen and cottonwood, are widely distributed throughout the northern hemisphere from subtropical to boreal forests (Brawdshaw et al., 2000), and China is one of the most important poplar distribution areas (Wan et al., 2013). *Populus* species play important roles in ecosystems and serve as model organisms for basic research in molecular biology and genetics, as well as in plant domestication and conservation (Kuzovkina and Vietto, 2014), mainly due to their fast growth rates, easy vegetative propagation, numerous wood uses, and small genome sizes (Braatne et al., 1992; Stettler et al., 1996; Cronk, 2005; Hamzeh et al., 2006).

Eckenwalder (1996) classified the genus *Populus* into 29 species grouped into six sections based on important morphological characteristics: persistence of the floral disk, presence or absence of marked foliar heteroblasty, overall leaf shape, distribution and shape of foliar teeth, number of carpels and flattened vs. round petiole. Section *Abaso* comprises a single species, *P. mexicana*; section *Aigeiros* contains three species and widely distributed in Europe and North America; section *Leucoides* shows a limited geographic distribution, with two species in China and the remaining species in southeastern United States; section *Turanga* contains three species and is geographically restricted to Central and West Asia and North Africa; sections *Tacamahaca* and *Populus* are relatively large, comprising nine and ten species, respectively (Hamzeh et al., 2006). However, the phylogenetic affinities between sections and the position of several taxa within these sections remain controversial. Many of the *Populus* species within a section, as well as between sections, are cross-compatible with each other (Hamzeh et al., 2006; Fladung and Schroeder, 2010). For example, species of the sections *Aigeiros* and *Tacamahaca* are sexually compatible, and natural hybridization occurs among several species of these sections (Zsuffa, 1975; Rajora and Zsuffa, 1984). Because of interspecific hybridization and high levels of morphological variation among poplars, the number of *Populus* species currently described in the literature ranges from 22 to 85, and hundreds of *Populus* hybrids and cultivars exist (Eckenwalder, 1977a,b, 1996; Dickmann and Stuart, 1983; Wang and Gilbert, 2007; Wan et al., 2013). Discrepancies in the total number of *Populus* species could be attributed to the misinterpretation of some hybrids and difficulties involved in delineating species boundaries (Hamzeh and Dayanandan, 2004).

For the assessment of genetic relationships among *Populus* species, different methods have been applied. A phylogenetic analysis of poplars using 76 morphological traits of leaves, flowers, fruits, and inflorescences supported the monophyly of all sections except for *Tacamahaca*, which resolved into two paraphyletic groups (Eckenwalder, 1996). *ITS* sequences were used in a study of fifteen representative species from five sections to resolve the relationships among the species in the genus *Populus* (Shi et al., 2001). The results showed that *Populus* is a monophyletic group and can be divided into two main clades: section *Leuce* and the remaining sections. Another phylogenetic study, based on three non-coding regions of cpDNA (intron of *trnL* and intergenic regions of *trnT-trnL* and *trnL-trnF*) and two nuclear rDNAs (*ITS1* and *ITS2*), showed polyphyletic relationships among species in the sections *Tacamahaca* and *Aigeiros* (Hamzeh and Dayanandan, 2004). The phylogenetic tree of seventeen species or hybrids from four sections in *Populus* based on the chloroplast marker *trnL-F* showed that the *Populus* section formed a separate clade, while sections *Tacamahaca* and *Turanga* could not be clearly separated, and *P. szechuanica* var. *tibetica* formed a weakly supported clade with the other species of section *Tacamahaca* (Wei et al., 2010). Moreover, based on the different combinations of plastid genomes (*rbcL-a, psbI-psbK, psbA-trnH*, and *trnL-trnF*), the phylogenetic relationship of 63 *Populus* individuals representing 32 species from five sections suggests that sections *Populus* and *Leucoides* formed a separate clade, while section *Tacamahaca* was divided into two groups, albeit with very weak bootstrap support (Yun et al., 2015). Although previous molecular systematic studies have been conducted, they were unable to solve the phylogenic relationships of *Populus* well. To reconstruct a more reliable phylogeny of *Populus*, it was necessary to sample more taxa, as well as to use more variable markers and genome sequences to resolve the phylogenetic problems at a lower taxonomic level. In addition, the most recent molecular studies of *Populus* have not explicitly analyzed morphology, therefore, a combined analysis including both molecular data and morphological data. Here, 24 newly sequenced *Populus* plastomes, sixteen *Populus*, fifteen *Salix*, and four previously reported plastomes and one partial *Chosenia* chloroplast genome sequence, all of which represent seven genera of the Salicaceae family, were used to reconstruct phylogenetic relationships.

## Materials and Methods

### Plant Materials

Twenty-four taxa, *P. adenopoda, P. alba, P. bolleana, P. cathayana, P. davidiana, P. deltoides, P. euphratica, P. gonggaensis, P. haoana, P. hopeiensis, P. kangdingensis, P. lasiocarpa, P. pseudoglauca, P. qamdoensis, P. rotundifolia* var. *duclouxiana, P. schneideri, P. tomentosa, P. tomentosa* narrow crown (*P. tomentosa* (NC)), *P. simonii, P. deltoides* × *nigra, P. szechuanica, P. szechuanica* var. *tibetica, P. trinervis*, and *P. yunnanensis*, representing the genus *Populus* of the family Salicaceae, were sampled following the taxonomy system of Eckenwalder (1996) and the Flora of China (Fang et al., 1999; Wu, 1999). We collected healthy, tender and fresh leaves from adult plants of target species. Details of the samples are given in Table 1. The voucher herbarium specimens for the 24 sampled *Populus* taxa were deposited at Southwest Forestry University (SWFU), Kunming, China.

**Table 1:** List of samples, voucher collection information and GenBank accession numbers.

No.	Taxa	Source	Voucher	NCBI numbers
1	*P. adenopoda*	Hunan, China	XA1 (SWFU)	MK267321
2	*P. alba*	Beijing, China	YB1 (SWFU)	MK267320
3	*P. bolleana*	Gansu, China	XJ1 (SWFU)	MK267319
4	*P. cathayana*	Sichuan, China	QY33-5 (SWFU)	MK267318
5	*P. davidiana*	Yunnan, China	SY1 (SWFU)	MK267317
6	*P. deltoides*	Yunnan, China	MH1 (SWFU)	MK267316
7	*P. deltoides* × *nigra*	Yunnan, China	SJ1 (SWFU)	MK267315
8	*P. euphratica*	Beijing, China	HY6-3-9 (SWFU)	MK267314
9	*P. gonggaensis*	Sichuan, China	GY1 (SWFU)	MK267313
10	*P. haoana*	Yunnan, China	DQ1 (SWFU)	MK267312
11	*P. hopeiensis*	Shanxi, China	HBY1 (SWFU)	MK267311
12	*P. kangdingensis*	Sichuan, China	KD1 (SWFU)	MK267310
13	*P. lasiocarpa*	Sichuan, China	EU1 (SWFU)	MK267309
14	*P. pseudoglauca*	Sichuan, China	UY1 (SWFU)	MK267308
15	*P. qamdoensis*	Tibet, China	CD1 (SWFU)	MK267307
16	*P. rotundifolia* var. *duclouxiana*	Yunnan, China	QX1 (SWFU)	MK267306
17	*P. schneideri*	Sichuan, China	XN1 (SWFU)	MK267305
18	*P. simonii*	Gansu, China	XY1 (SWFU)	MK267304
19	*P. szechuanica*	Sichuan, China	CY1 (SWFU)	MK267303
20	*P. szechuanica* var. *tibetica*	Tibet, China	ZCY1 (SWFU)	MK267302
21	*P. tomentosa*	Yunnan, China	M1 (SWFU)	MK267301
22	*P. tomentosa* (NC)	Hebei, China	ZG4 (SWFU)	MK192135
23	*P. trinervis*	Sichuan, China	SM1 (SWFU)	MK267300
24	*P. yunnanensis*	Yunnan, China	DY1 (SWFU)	MK267299

### DNA Extraction and Assembly

Total genome DNA was extracted with the Ezup plant genomic DNA prep Kit (Sangon Biotech, Shanghai, China). DNA samples were properly deposited in Key Laboratory of State Forestry Administration on Biodiversity Conservation in Southwest China, SWFU, Kunming. Total DNA was used to generate libraries with an average insert size of 400 bp and sequences using the Illumina HiSeqX platform. Approximately 15.0 GB of raw data were generated with 150 bp paired-end read lengths. Then, the raw data were used to assemble the complete chloroplast genome using GetOrganelle software (Jin et al., 2018) with *P. trichocarpa* as the reference. Genome annotation was performed with the program Geneious R8 (Biomatters Ltd., Auckland, New Zealand), and the start and stop codons were manually adjusted by comparison with the cp genome of *P. trichocarpa*. The tRNA genes were further confirmed using online tRNAscan-SE web servers (Schattner et al., 2005). The gene map of the annotated *Populus* chloroplast genome was drawn by OGdraw online (Lohse et al., 2007).

### Sliding Window Analysis of the Plastomes

From NCBI GenBank, sixteen plastome sequences of *Populus* [*P. angustifolia* (GenBank accession number: MG262345), *P. balsamifera* (GenBank accession number: KJ664927), *P. fremontii* (GenBank accession number: KJ664926), *P. ilicifolia* (GenBank accession number: KX421095), *P. koreana* (GenBank accession number: MG262348), *P. laurifolia* (GenBank accession number: MG262350), *P. mexicana* (GenBank accession number: MG232351), *P. nigra* (GenBank accession number: KX377117), *P. pruinosa* (GenBank accession number: MG262355), *P. qiongdaoensis* (GenBank accession number: KX534066), *P. rotundifolia* (GenBank accession number: KX425853), *P. tremula* (GenBank accession number: KP861984), *P. tremula* × *alba* (GenBank accession number: KT780870), *P. trichocarpa* (GenBank accession number: EF489041), *P. wilsonii* (GenBank accession number: MG214781)], *P. xiangchengensis* (GenBank accession number: MH910611) reported in our previous study (Zong et al., 2019), and fifteen plastomes of *Salix* [*S. arbutifolia* (GenBank accession number: KX781246), *S. babylonica* (GenBank accession number: KT449800), *S. chaenomeloides* (GenBank accession number: MG262362), *S. hypoleuca* (GenBank accession number: MG262363), *S. interior* (GenBank accession number: KJ742926), *S. magnifica* (GenBank accession number: MG262364), *S. minjiangensis* (GenBank accession number: MG262365), *S. oreinoma* (GenBank accession number: MF189168), *S. paraplegia* (GenBank accession number: MG262366), *S. purpurea* (GenBank accession number: KP019639), *S. rehderiana* (GenBank accession number: MG262367), *S. rorida* (GenBank accession number: MG262368), *S. suchowensis* (GenBank accession number: KM983390), *S. taoensis* (GenBank accession number: MG262369) and *S. tetrasperma* (GenBank accession number: MF189169)] were collected. MAFFT version 7 software was used to align the 40 plastome sequences of *Populus*, fifteen plastomes of *Salix*, and all 55 plastomes, respectively (Katoh and Standley, 2013). After manual adjustment with MEGA 5 (Tamura et al., 2011), sliding window analysis was performed to assess the variability (Pi) of the whole plastomes in DnaSP version 5 software (Librado and Rozas, 2009). The window length was set to 600 bp, and the step size was set to 200 bp.

### Phylogenetic Analysis

To estimate phylogenetic relationships within the *Populus*, 59 taxa with available complete plastid genomes and one taxa (*Chosenia arbutifolia*) with seven combined chloroplast fragments [*matK* (GenBank accession number: EU790701), *ndhF* (GenBank accession number: AY757181), *rbcL*-*atpB* (GenBank accession number: FJ788535), *trnL* (GenBank accession number: GQ244791), *trnD-trnT* (GenBank accession number: FJ788620), *rbcL* (GenBank accession number: AB012776), and *trnL-F* (GenBank accession number: AY757062)] were compared, with 40 taxa from *Populus*, fifteen taxa from *Salix* and four taxa from four genera that are members of the Salicaceae family, *Flacourtia indica* (GenBank accession number: MG262341), *Idesia polycarpa* (GenBank accession number: KX229742), *Itoa orientalis* (GenBank accession number: MG262342), and *Poliothyrsis sinensis* (GenBank accession number: MG262343) were sampled as outgroups. The 40 *Populus* genomes comprised the 24 new plastomes and sixteen previously published complete plastomes adopted from the NCBI (Chen et al., 2016; Wang et al., 2016; Zheng et al., 2016; Zong et al., 2019). The complete genome matrix was aligned using MAFFT version 7 software (Katoh and Standley, 2013) and then manually edited using MEGA 5 (Tamura et al., 2011). The matrix of 60 chloroplast genome sequences included 180,546 characters, 4,425 of which were parsimary informative sites. Based on these parsimary informative sites, we performed maximum likelihood and Bayesian inference analyses. A maximum likelihood method for phylogenetic analysis was performed based on the GTR+I+G model in the RAxML version 8 with 1000 bootstrap replicates (Stamatakis, 2014). Maximum likelihood (ML) bootstrap support values (BS) ≥70% were considered well supported, and ML BS <50% were considered poorly supported or unresolved. Bayesian inference (BI) was performed using MrBayes 3.1.2 (Ronquist and Huelsenbeck, 2003). jModelTest 2.0 (Darriba et al., 2012) was used to determine the best-fitting model for each dataset based on the Akaike information criterion (AIC), and the optimal model “GTR+F+R5” (freqA = 0.313, freqC = 0.187, freqG = 0.180, freqT = 0.320, R(a) [AC] = 0.949, R(b) [AG] = 1.621, R(c) [AT] = 0.916, R(d) [CG] = 0.552, R(e) [CT] = 1.621, R(f) [GT] = 1.000) was selected. The Markov chain Monte Carlo (MCMC) algorithm was run for 1000,000 generations, and a burn-in of 25% was used for the analysis. Internodes with posterior probability (PP) values ≥0.95 were considered statistically significant.

### Morphological Analysis

The classification of *Populus* was based on the Flora of China (Fang et al., 1999; Wu, 1999), and 40 taxa representing the genus of *Populus* were sampled. Morphological information was collected from personal observations of live plants, herbarium specimens, and the available literature (Fang et al., 1999; Argus, 2010), which were evaluated for eighteen morphological characters: (1) bark smoothness; (2) bark color; (3) branchlet; (4) bud; (5) bud size; (6) bud viscid; (7) petiole vs. leaf; (8) petiole shape; (9) petiole; (10) petiole glands; (11) leaf presence in short branchlet; (12) leaf apex; (13) leaf margin; (14) leaf base; (15) leaf; (16) leaf in inflorescence; (17) male anther length; and (18) male anther tip (Table 2), and a matrix of eighteen discrete morphological characters was constructed for all of the taxa investigated (Table 3).

**Table 2:** Character coding for morphological analysis.

No.	Characters	Character states					
1	Bark smoothness	Furrowed (1)	Shallowly furrowed (2)	Smooth (3)			
2	Bark color	Grayish green (1)	Grayish white or grayish green (2)	Grayish (3)	Dark gray (4)	Grayish white (5)	
3	Branchlet	Glabrous to pubescent (1)	Glabrous or pubescent (2)	Pubescent (3)	Glabrous (4)		
4	Bud	Glabrous or pubescent (1)	Pubescent (2)	Glabrous (3)			
5	Bud size	Middle (1)	Large (2)	Small (3)			
6	Bud viscid	Absent (0)	Present (1)				
7	Petiole vs. leaves	Short or equal (1)	Equal (2)	Long or equal (3)	Short (4)		
8	Petiole shape	Micro flat (1)	Terete (2)	Flat (3)			
9	Petiole	Glabrous (0)	Pubescent (1)				
10	Petiole glands	Absent or present (1)	Present (2)	Absent (3)			
11	Leaf in short branchlet	Ovate (1)	Deltoid-ovate (2)	Oval (3)	Ovate-lanceolate (4)		
12	Leaf apex	Acute (1)	Acuminate (2)	Blunt (3)			
13	Leaf margin	Wavy dentate (1)	Coarsely dentate (2)	Crenature (3)	Glandular crenate (4)	Glandular serrate (5)	Wavy entire (6)
14	Leaf base	Round (1)	Cuneate (2)	Cordate (3)	Broadly cuneate (4)		
15	Leaf	Pubescent to glabrous (1)	Pubescent abaxially (2)	Pubescent adaxially (3)	Glabrous (4)		
16	Leaf in inflorescence	No or small leaf (1)	Present leaf (2)	No leaf (3)			
17	Male anther length	Short (0)	Long (1)				
18	Male anther tip	Cuneate (0)	Apicule (1)				

**Table 3:** Data matrix of morphological characters in the study.

Taxa	1	2	3	4	5	6	7	8	9	10	11	12	13	14	15	16	17	18
*P. adenopoda*	3	5	1	3	3	1	4	3	1	2	3	2	1	2	1	3	0	0
*P. alba*	3	5	3	2	3	0	1	3	1	3	3	3	2	2	2	3	0	0
*P. angustifolia*	2	3	4	3	1	2	4	2	0	3	4	3	3	1	4	3	0	0
*P. balsamifera*	1	4	4	3	1	2	4	2	0	1	1	3	3	4	4	3	0	0
*P. bolleana*	3	5	3	2	3	0	1	3	1	3	3	3	2	2	2	3	0	0
*P. cathayana*	2	4	4	3	2	2	4	2	0	3	1	2	4	4	4	3	0	0
*P. davidiana*	3	2	1	3	3	1	1	3	0	2	3	3	1	2	1	3	0	0
*P. deltoides*	1	5	2	1	1	2	2	3	0	1	2	2	4	2	4	3	0	0
*P. deltoides × nigra*	1	5	2	1	3	2	2	3	0	1	2	2	3	2	4	3	0	0
*P. euphratica*	2	4	4	2	3	0	2	1	0	2	1	2	1	4	1	1	1	1
*P. fremontii*	2	5	2	2	1	2	4	3	0	3	2	2	3	2	4	3	0	0
*P. gonggaensis*	1	4	3	3	2	2	4	2	1	2	1	2	4	1	4	3	1	1
*P. haoana*	1	3	3	2	2	2	4	2	1	3	1	2	5	3	4	3	0	0
*P. hopeiensis*	3	5	3	2	3	0	1	3	1	3	3	3	2	2	2	3	0	0
*P. ilicifolia*	2	3	4	2	3	0	4	1	0	2	4	1	2	4	4	2	1	1
*P. kangdingensis*	1	3	3	3	2	2	4	2	1	3	1	1	5	4	4	3	0	0
*P. koreana*	2	4	4	2	2	2	4	2	1	3	4	3	4	4	4	3	0	0
*P. lasiocarpa*	1	4	1	2	2	1	4	2	1	2	1	2	1	3	2	3	1	1
*P. laurifolia*	2	3	3	2	2	2	4	2	1	3	4	1	4	4	4	3	0	0
*P. mexicana*	1	5	4	3	1	1	3	1	0	3	2	2	3	2	4	1	1	1
*P. nigra*	2	5	2	1	3	2	2	3	0	1	2	2	3	2	4	3	0	0
*P. pruinosa*	2	5	3	2	3	0	2	1	0	2	3	3	6	4	4	1	1	1
*P. pseudoglauca*	1	4	3	2	2	2	4	2	1	2	1	2	4	3	2	3	1	1
*P. qamdoensis*	1	3	1	2	2	2	4	2	1	3	1	2	3	1	3	3	0	0
*P. qiongdaoensis*	2	3	1	2	3	0	4	3	1	2	3	2	1	2	2	3	0	0
*P. rotundifolia*	3	5	1	2	3	2	3	3	0	3	3	2	1	2	4	3	0	0
*P. rotundifolia* var. *duclouxiana*	3	5	1	2	3	2	4	3	0	2	3	2	1	2	4	3	0	0
*P. schneideri*	1	3	3	3	2	2	4	2	1	3	1	2	5	1	4	3	0	0
*P. simonii*	2	4	4	3	2	2	4	2	0	3	1	1	5	4	4	3	0	0
*P. szechuanica*	1	3	4	3	2	2	4	2	0	3	4	2	5	4	4	3	0	0
*P. szechuanica* var. *tibetica*	1	3	4	2	2	2	4	2	1	3	4	2	5	4	1	3	0	0
*P. tomentosa*	3	2	3	2	3	0	4	3	1	2	3	2	2	2	2	3	0	0
*P. tomentosa* (NC)	3	2	3	2	3	0	4	3	1	2	3	2	2	2	2	3	0	0
*P. tremula*	3	1	2	3	3	1	2	3	0	2	3	3	1	2	4	3	0	0
*P. tremula × alba*	3	2	2	3	3	1	2	3	0	2	3	3	1	2	4	3	0	0
*P. trichocarpa*	1	5	3	1	1	2	4	2	0	1	1	2	3	4	4	3	0	0
*P. trinervis*	2	3	4	3	2	2	4	2	0	3	1	2	4	4	3	3	0	0
*P. wilsonii*	1	4	4	3	2	1	1	2	0	2	1	3	4	3	1	3	1	1
*P. xiangchengensis*	1	3	3	2	2	2	4	2	1	3	1	2	3	1	4	3	0	0
*P. yunnanensis*	1	3	4	3	2	2	4	2	0	3	4	2	4	4	4	3	0	0

To evaluate the morphological characters supporting relationships based on molecular data and to evaluate whether the investigated characters were phylogenetically conserved at the level of the whole phylogeny, we calculated mean Pagel’s lambda (λ) (Pagel, 1999) and Blomberg’s *K* values (Garland et al., 1992) at the species level for each character, thus obtaining phylogenetic information. Both indices assume the classic Brownian motion (BM) evolutionary model, with values varying from zero to one for λ and from zero to higher than one for *K*. λ values close to zero indicate there is no phylogenetic signal (the traits have evolved independently of phylogeny, and the traits of close relatives are not more similar than those of distant relatives), and λ values close to one indicate trait evolution according to BM. *K-*values close to zero indicate the phylogenetic signal is weaker than expected from the BM model of character evolution (low levels of phylogenetic character conservation). *K-*values close to or higher than one indicate a strong phylogenetic signal (Molina-Venegas and Rodríguez, 2017).

The significance of phylogenetic signals was determined by shuffling species’ character values (999 times) across the tips of the phylogenetic tree and comparing the resulting *K-*values to those computed from the observed character data (Eichenberg et al., 2015), whereas the statistical significance of λ was assessed based on a comparison with the likelihood of a model that assumes complete phylogenetic independence (Pagel, 1999). The Bayesian tree based on the complete chloroplast genome sequences provided the standard tree topology. Phylogenetic signal analyses were carried out using the routines provided in the picante package available for R (Kembel et al., 2010).

## Results

### Range of Variation in Different Plastomes of *Populus*

**FIGURE 1:**
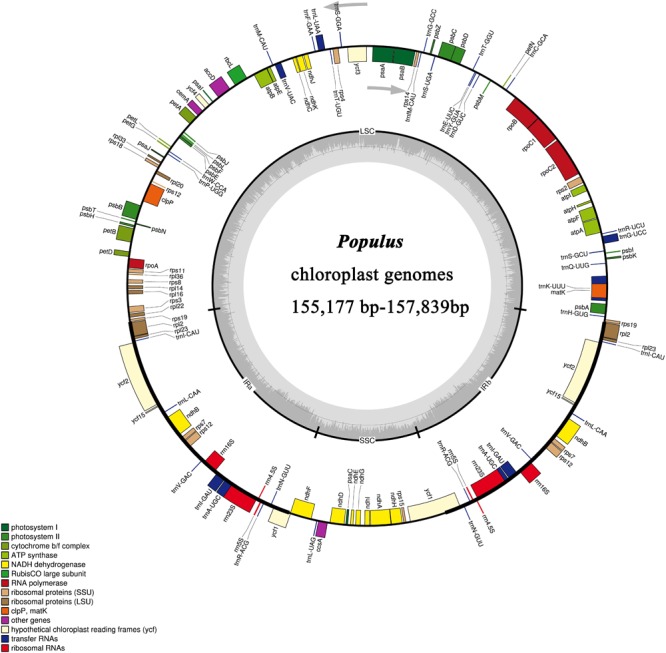
Gene map of 24 *Populus* chloroplast genomes. Genes inside the circle are transcribed clockwise and genes outside the circle are transcribed counter clockwise.

In this study, we determined the structure characteristics and gene contents of the complete plastid genomes of 24 *Populus* taxa. The cp genome lengths of the 24 *Populus* taxa ranged from 155,177 bp for *P. rotundifolia* var. *duclouxiana* to *P. euphratica* for 157,839 bp (Figure 1). All of these assembled into single circular, double-stranded DNA sequences, presenting a typical quadripartite structure, including one LSC with a length of 85,858 bp (*P. euphratica*) to 84,450 bp (*P. rotundifolia* var. *duclouxiana*), one SSC with a length of 16,421 bp (*P. szechuanica* var. *tibetica*) to 16,879 bp (*P. hopeiensis*), and a pair of IR with a length of 26,903 bp (*P. hopeiensis*) to 27,672 bp (*P. cathayana*) (Table 4). All 24 chloroplast genomes contained 130 genes, of which 112 were unique and eighteen were duplicated in IR regions. Among the 112 unique genes, 78 protein-coding genes, 30 tRNA genes and four rRNA genes were identified. Six protein-coding genes (*atpF, ndhA, ndhB, petB, rpl2*, and *rpoC1*) and six tRNA genes (*trnA-UGC, trnG-UCC, trnI-GAU, trnK-UUU, trnL-UAA*, and *trnV-UAC*) had a single intron, while three protein-coding genes (*ycf3, clpP*, and *rps12*) possessed two introns (Supplementary Table S1). The GC content of these plastomes ranged from 36.5% in *P. euphratica* to 36.8% in *P. tomentosa* (NC), *P. tomentosa, P. alba, P. bolleana, P. davidiana, P. rotundifolia* var. *duclouxiana*, and *P. hopeiensis*. The GC content of the LSC region (34.2–34.6%) and SSC region (30.4–30.7%) was lower than that of the IR regions (41.9–42.3%). The high GC percentage of the IR regions was possibly due to the presence of four rRNA genes in these regions.

**Table 4:** Summary of complete chloroplast genomes for 24 *Populus* species.

Taxa	Length/bp	LSC/bp	IR/bp	SSC/bp	Total genes	CDS	tRNA	rRNA	Total GC content (%)	GC content in LSC (%)	GC content in IR (%)	GC content in SSC (%)
*P. adenopoda*	156,537	84,727	27,625	16,560	130	85	37	8	36.7	34.5	42.0	30.5
*P. alba*	156,446	84,685	27,610	16,541	130	85	37	8	36.8	34.6	42.0	30.5
*P. bolleana*	156,278	84,492	27,624	16,538	130	85	37	8	36.8	34.6	42.0	30.5
*P. cathayana*	156,789	84,851	27,672	16,594	130	85	37	8	36.7	34.6	41.9	30.6
*P. davidiana*	155,415	84,641	26,955	16,864	130	85	37	8	36.8	34.5	42.3	30.5
*P. deltoides*	156,957	85,096	27,649	16,563	130	85	37	8	36.7	34.5	41.9	30.6
*P. deltoides × nigra*	156,957	85,096	27,649	16,563	130	85	37	8	36.7	34.5	41.9	30.6
*P. euphratica*	157,839	85,858	27,666	16,649	130	85	37	8	36.5	34.2	41.9	30.6
*P. gonggaensis*	156,466	84,813	27,570	16,513	130	85	37	8	36.7	34.5	42.0	30.7
*P. haoana*	156,523	84,788	27,620	16,495	130	85	37	8	36.7	34.5	42.0	30.5
*P. hopeiensis*	155,367	84,682	26,903	16,879	130	85	37	8	36.8	34.5	42.3	30.4
*P. kangdingensis*	156,523	84,788	27,620	16,495	130	85	37	8	36.7	34.5	42.0	30.5
*P. lasiocarpa*	156,525	84,834	27,620	16,451	130	85	37	8	36.7	34.5	42.0	30.6
*P. pseudoglauca*	156,512	84,777	27,620	16,495	130	85	37	8	36.7	34.5	42.0	30.5
*P. qamdoensis*	156,526	84,793	27,619	16,495	130	85	37	8	36.7	34.5	42.0	30.5
*P. rotundifolia* var. *duclouxiana*	155,177	84,450	26,931	16,865	130	85	37	8	36.8	34.5	42.3	30.5
*P. schneideri*	156,513	84,778	27,620	16,495	130	85	37	8	36.7	34.5	42.0	30.5
*P. simonii*	156,475	84,750	27,612	16,501	130	85	37	8	36.7	34.5	42.0	30.5
*P. szechuanica*	156,444	84,701	27,620	16,503	130	85	37	8	36.7	34.5	42.0	30.5
*P. szechuanica* var. *tibetica*	156,518	84,809	27,644	16,421	130	85	37	8	36.7	34.5	41.9	30.6
*P. tomentosa*	156,446	84,685	27,610	16,541	130	85	37	8	36.8	34.6	42.0	30.5
*P. tomentosa* (NC)	156,446	84,685	27,610	16,541	130	85	37	8	36.8	34.6	42.0	30.5
*P. trinervis*	156,465	84,812	27,570	16,513	130	85	37	8	36.7	34.5	42.0	30.7
*P. yunnanensis*	156,506	84,770	27,621	16,494	130	85	37	8	36.7	34.5	42.0	30.5

### IR Expansion and Contraction of *Populus*

**FIGURE 2:**
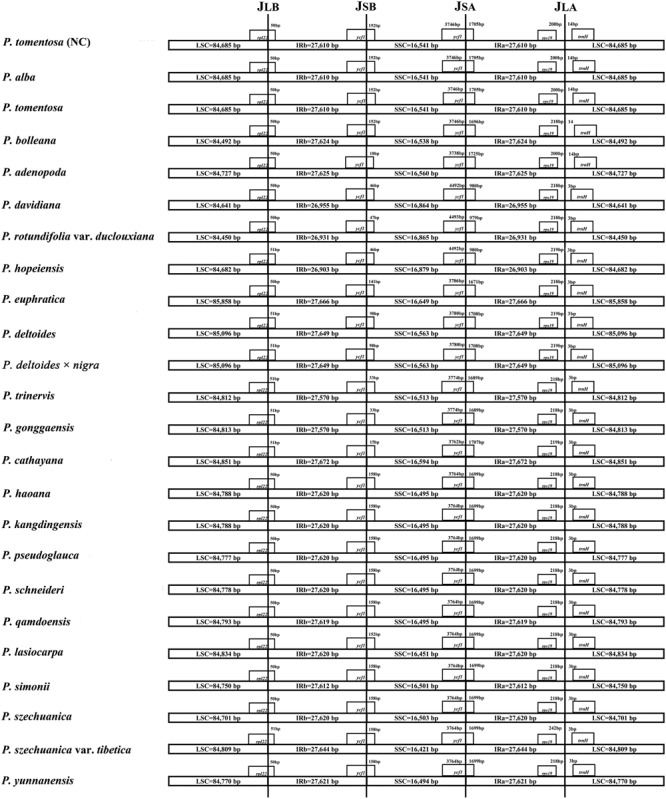
Comparison of the borders of the LSC, SSC, and IR regions of 24 *Populus* plastomes.

The expansion and contraction of the IR region and the single copy (SC) boundary regions is considered a primary mechanism causing the length variation of angiosperm cp genomes (Kim and Lee, 2005). Although the overall genomic structure, including gene number and gene order, was well conserved among the *Populus* plastid genomes, these genomes exhibited obvious differences in the IR/SC boundary regions. The IR regions of the *Populus* plastid genomes ranged from 26,903 bp (*P. hopeiensis*) to 27,672 bp (*P. cathayana*) in size, and two complete or fragmented copies of *rpl22* and *ycf1* were located at the boundaries between the LSC or SSC regions and IRs regions in the 24 *Populus* plastomes. The full lengths of the *rpl22* and *ycf1* genes were 399 and 5442 to 5472 bp, respectively. The *rpl22* gene crossed the IR-LSC boundary, with only 1 bp variation in sequence length among the *Populus* plastomes. The gene *trnH* in the LSC region contracted by 14 bp from the junction region of the IR-LSC boundary in four taxa, i.e., *P. alba, P. bolleana, P. tomentosa*, and *P. tomentosa* (NC), while the other taxa contracted by 3 bp. In addition, *rps19* in the IRa region also contracted by a different number of bases (200–242 bp) among taxa. Gene *ycf1* in the IRb region extended from 15 to 170 bp, whereas gene *ycf1* in the IRa region extended 979 to 1725 bp (Figure 2).

### Comparative Analysis of the *Populus* Chloroplast Genome

**FIGURE 3:**
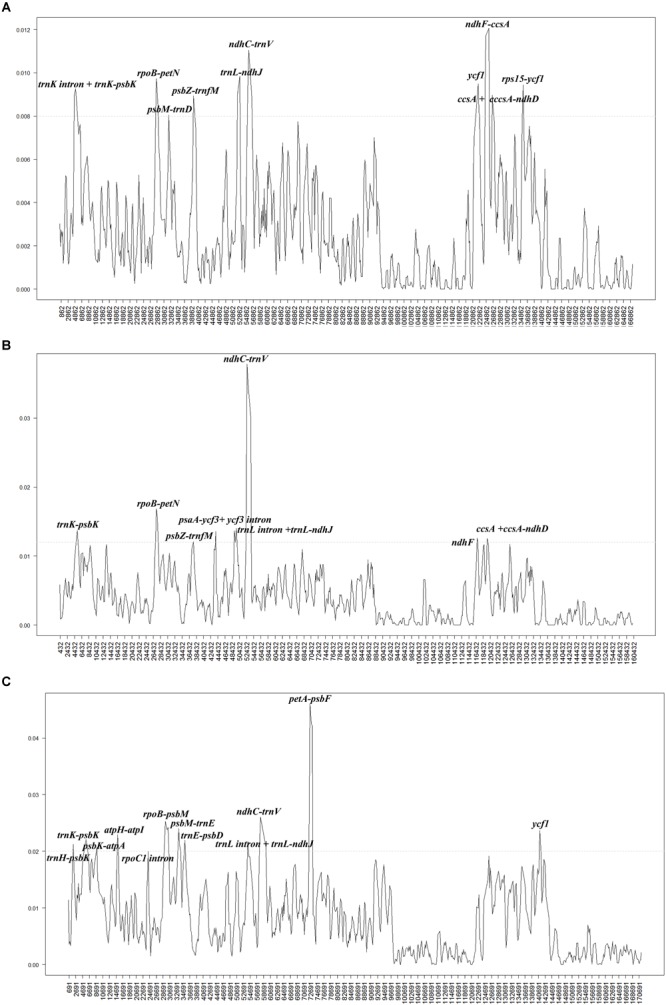
Comparison of the nucleotide variability (Pi) values of the plastomes: **(A)** 40 plastomes of *Populus*, **(B)** fifteen plastomes of *Salix*, and **(C)** 55 plastomes of *Populus* and *Salix*. The genetic divergence was calculated with DnaSP 5.0 software (window length: 600 bp and step size: 200 bp). *X*-axis: position of the midpoint of a window, *Y*-axis: nucleotide diversity of each window.

To investigate the levels of sequence divergence, the nucleotide variability (Pi) values within 600 bp in 40 *Populus* plastomes were calculated with DnaSP software. The nucleotide variability values within these 600 bp varied from 0 to 0.00713, with a mean of 0.00359, among the 40 plastomes (Supplementary Table S2). The pairwise nucleotide divergence between two of the 40 plastomes varied from 0.000 to 0.003 (Supplementary Table S3). Both results indicate high sequence similarity across the 40 chloroplast genomes, suggesting that the plastomes of *Populus* are highly conserved. All aligned sequences demonstrate surprising low divergences, with only ten regions displaying high variation (Pi > 0.008). The ten most dissimilar regions of the 40 plastomes were *trnK intron* + *trnK-psbK, rpoB-petN, psbM-trnD, psbZ-trnfM, trnL-ndhJ, ndhC-trnV, ycf1, ndhF-ccsA, ccsA* + *ccsA-ndhD*, and *rps15-ycf1* (Figure 3A). Among these regions, six (*trnK intron* + *trnK-psbK, rpoB-petN, psbM-trnD, psbZ-trnfM, trnL-ndhJ*, and *ndhC-trnV*) were located in the LSC region, and four were located in the SSC region (*ycf1, ndhF-ccsA, ccsA* + *ccsA-ndhD*, and *rps15-ycf1*). In addition, eight hotspot regions were found (Pi > 0.012) in the comparison of fifteen *Salix* plastomes. These eight hotspot regions were *trnK- trnQ, rpoB-petN, psbZ-trnfM, psaA-ycf3* + *ycf3 intron, trnL intron* + *trnL-ndhJ, ndhC-trnV, ndhF, ccsA* + *ccsA-ndhD* (Figure 3B). Twelve hotspot regions (*trnH-trnK, psbK-atpA, atpH-atpI, rpoC1 intron, rpoB-psbM, psbM-trnE, trnE-psbD, trnL intron* + *trnL-ndhJ, ndhC-trnV, petA-psbF*, and *ycf1*) were shared between *Populus* and *Salix* (Pi > 0.02) (Figure 3C).

### Phylogenomic Analysis

**FIGURE 4:**
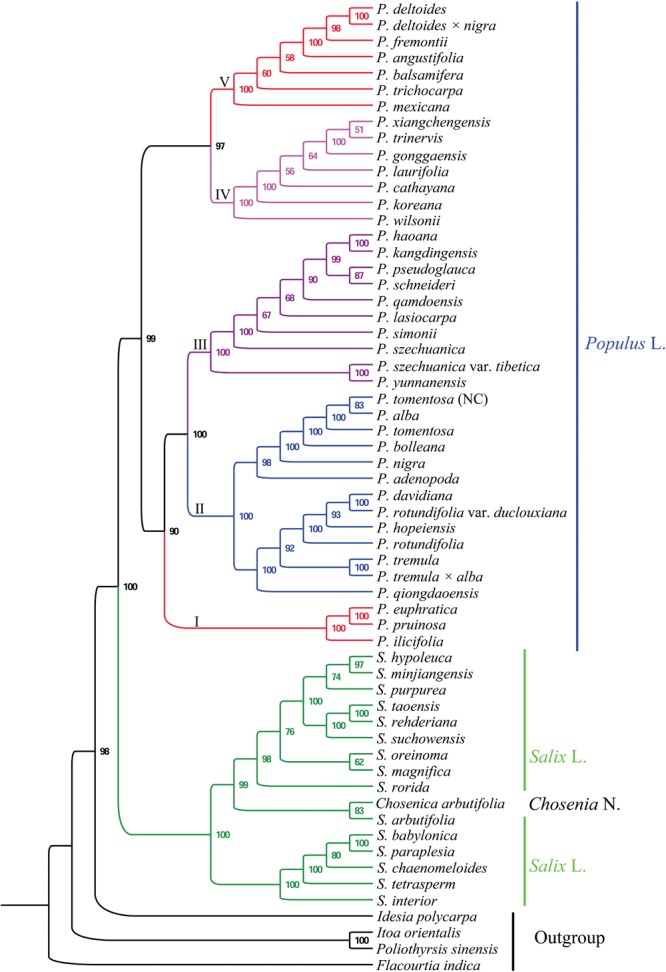
Phylogenetic tree reconstructions of *Populus* using maximum likelihood (ML) based on whole chloroplast genome sequences.

**FIGURE 5:**
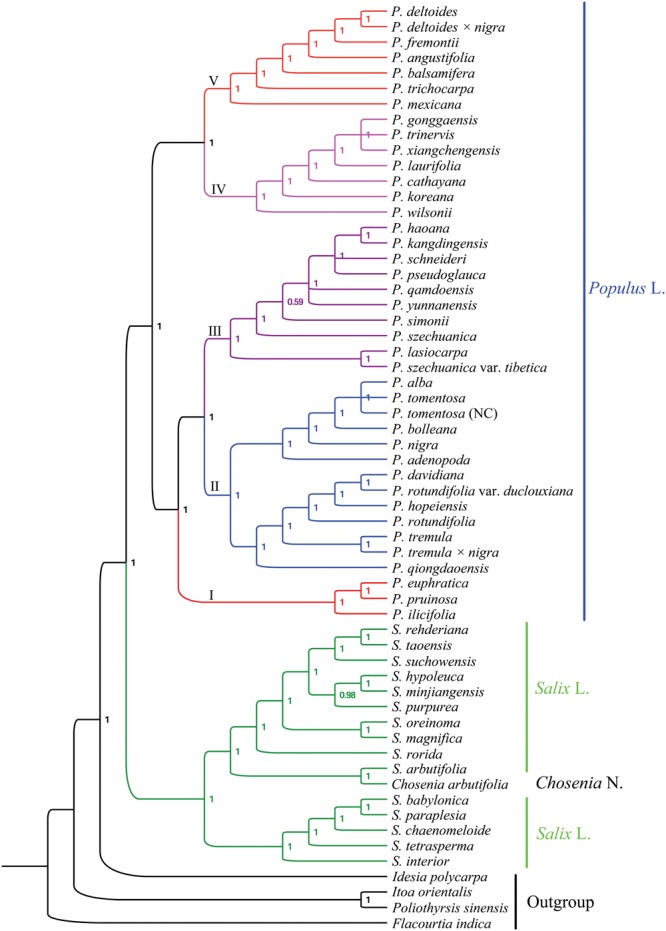
Phylogenetic tree reconstructions of *Populus* using Bayesian inference (BI) based on whole chloroplast genome sequences.

The matrix of the complete plastomes was used to reconstruct a phylogenetic tree of *Populus* and *Salix* (Figures 4, 5). *Populus* and *Salix* were two independent main clades (BS = 100% and PP = 1). *C. arbutifolia* was nested within the genus *Salix*, while the sisterhood of *Populus* and *Salix* was highly supported. Five well-supported clades were recovered within *Populus* (BS > 90% and PP = 1). Clade I (BS = 90% and PP = 1) comprised three taxa: *P. euphratica, P. ilicifolia* and *P. pruinosa*. Clade II (BS = 100% and PP = 1) contained thirteen plastomes for the taxa *P. adenopoda, P. alba, P. bolleana, P. davidiana, P. hopeiensis, P. nigra, P. qiongdaoensis, P. rotundifolia, P. rotundifolia* var. *duclouxiana, P. tremula, P. tremula* × *alba, P. tomentosa*, and *P. tomentosa* (NC). Clade III (BS = 100% and PP = 1) contained ten taxa: *P. haoana, P. kangdingensis, P. lasiocarpa, P. pseudoglauca, P. qamdoensis, P. schneideri, P. simonii, P. szechuanica, P. szechuanica* var. *tibetica*, and *P. yunnanensis*. Clade IV (BS = 97% and PP = 1) included the taxa *P. cathayana, P. gonggaensis, P. koreana, P. laurifolia, P. trinervis, P. wilsonii*, and *P. xiangchengensis*. The last clade (BS = 97% and PP = 1) was made up of *P. angustifolia, P. balsamifera, P. deltoides, P. deltoides* × *nigra, P. fremontii, P. mexicana*, and *P. trichocarpa*.

### Influence of Plant Phylogeny on Morphological Traits

Analysis of morphological characters showed that six characters, i.e., bark smoothness (λ = 8.340867e-01, *P* < 0.001; *K* = 1.0480, *P* < 0.001), bud size (λ = 9.076553e-01, *P* < 0.001; *K* = 0.72461, *P* < 0.001), petiole shape (λ = 9.817749e-01, *P* < 0.001; *K* = 1.40960, *P* < 0.001), leaf in inflorescence (λ = 6.208698e-01, *P* < 0.001; *K* = 0.58131, *P* = 0.08759), anther length (λ = 4.917771e+00, *P* < 0.001; *K* = 1.50515, *P* < 0.001), and anther tip (λ = 4.917771e+00, *P* < 0.001; *K* = 1.50515, *P* < 0.001), were highly congruent with the molecular phylogenies (Table 5).

**Table 5:** Six characters test well congruent with molecular phylogenies.

Characters	Lambda (λ)	Lambda_*P*	Blomb (*K*)	Blomb_*P*
1	8.340867e-01	0.00000	1.0480072	0.00009999
5	9.076553e-01	0.00010	0.7246106	0.00009999
8	9.817749e-01	0.00000	1.4095984	0.00009999
16	6.208698e-01	0.00006	0.5813071	0.08759124
17	4.917771e+00	0.00092	1.5051537	0.00009999
18	4.917771e+00	0.00092	1.5051537	0.00009999

## Discussion

### Comparative Analysis of the *Populus* Plastid Genomes

Complete plastid genomes are valuable sources of genetic markers for phylogenetic analyses because of their highly conserved genome structure (Provan et al., 2001; Chaney et al., 2016). Although the plastid genome generally has a nearly collinear gene order, changes in the genome, such as sequence inversion and gene expansion or contraction at the boundary of the SC and IR regions, occur over the course of evolution (Cho et al., 2015; Choi et al., 2016). Our results showed that the genome size, gene order, and compositions of the 24 newly sequenced *Populus* genomes were very similar with each other and with previously sequenced *Populus* plastid genomes; the genome sizes ranged from 155,177 to 157,839 bp (Chen et al., 2016; Wang et al., 2016; Zheng et al., 2016; Han et al., 2017). Among the genomes, three plastomes of *P. alba, P. tomentosa*, and *P. tomentosa* (NC), two plastomes of *P. haoana* and *P. kangdingensis*, two plastomes of *P. trinervis* and *P. xiangchengensis*, and two plastomes of *P. deltoides* and *P. deltoides* × *nigra*, each had the same length. All of the newly examined chloroplast genomes of *Populus* contained more AT than GC contents, with values ranging from 36.5 to 39.8%, and the GC contents of the IR regions were higher than those of the SC regions, possibly due to the presence of rRNA genes. Furthermore, we found that all 24 *Populus* plastomes encoded 85 protein-coding genes, 37 tRNA genes and eight rRNA genes, and eighteen of these genes were duplicated within the IRs. Moreover, the total intron numbers in the plastome were the same among the 24 *Populus* genomes, and in each plastome, twelve genes contained one intron, and three genes contained two introns.

The IR/LS boundary regions of the 24 complete *Populus* cp genomes were compared and showed slight differences in the junction positions. The *rpl22* gene crossed the LSC/IRb boundary with only one bp variation in sequence length among the *Populus* plastomes. The gene *trnH* was located in the LSC region of all genomes but contracted by 3 or 14 bp from the LSC/IRa junction region, with a contraction of 14 bp in the plastomes of *P. alba, P. bolleana, P. tomentosa*, and *P. tomentosa* (NC) and a contraction of 3 bp in the other taxa. Gene *ycf1* in the IRb region extended from 15 to 170 bp, whereas gene *ycf1* in the IRa region extended 979 to 1725 bp. The length changes in the truncated *ycf1* gene directly drive the contraction of the IR regions in the plastome of *Populus*.

### Low Sequence Divergence Among *Populus* and *Salix* Taxa

Although the length of the truncated *ycf1* gene varied and the divergence of the unbroken *ycf1* gene was high among the *Populus* taxa, the nucleotide variability of the whole plastome among these taxa was only 0.36%, which was approximately the same as the nucleotide variability of eight plastomes of *Alseodaphne gracilis, A. huanglianshanensis, A. semecarpifolia*, avocado, *Cinnamomum micranthum, Machilus balansae, Phoebe sheareri*, and *P. omeiensis* (0.30%) (Song et al., 2015, 2016, 2017, 2018) and was much lower than the sequence divergence among six *Cymbidium* species (3.70%) (Yang et al., 2013) and five *Epimedium* species (3.97%) (Zhang et al., 2016). In previous studies, *trnL-trnF, rbcL-a, psbI-psbK, psbA-trnH*, and *ITS* sequences did not completely resolve the phylogenetic relationships in the genus of *Populus* at the molecular level (Hamzeh and Dayanandan, 2004; Wei et al., 2010; Yun et al., 2015). Further work is still necessary to determine whether these ten variable loci (*trnK intron* + *trnK-psbK, rpoB-petN, psbM-trnD, psbZ-trnfM, trnL-ndhJ, ndhC-trnV, ycf1, ndhF-ccsA, ccsA* + *ccsA-ndhD*, and *rps15-ycf1*) could be used in *Populus* phylogenetic analyses or as excellent candidate markers for population genetic and phylogenetic analyses.

### Relationships in *Populus*

Most previous studies have used sequences from only one or more chloroplast loci in Salicaceae (Hamzeh and Dayanandan, 2004; Wei et al., 2010; Yun et al., 2015). However, the section delimitation and species relationships within *Populus* remain unclear. Our study included 60 plastid genomes for plants from seven genera of Salicaceae. All of these plastome sequences of *Populus* and related genera yielded a fully resolved tree, consistent with the Angiosperm Phylogeny Group’s most recent phylogeny, APG IV (Chase et al., 2002; Byng et al., 2016). In addition, two monophyletic genera: *Populus* and *Salix*, were strongly supported, and *Chosenia* taxa were nested within the genus *Salix*, which is consistent with previous studies (Leskinen and Alström-Rapaport, 1999; Chase et al., 2002). The results suggested that *Populus* is a monophyletic sister group to *Salix*.

**FIGURE 6:**
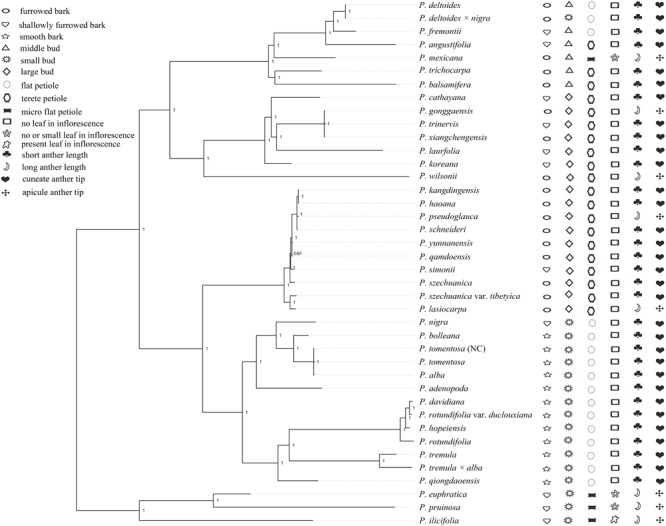
Morphological character reconstruction of *Populus* on the Bayesian phylogenetic tree.

Forty *Populus* taxa were separated into the following five clades in our study. Section *Turanga* in the study of Wu (1999) formed the first clade, including *P. euphratica, P. ilicifolia*, and *P. pruinosa*, in our phylogeny, which share the same character of micro flat petiole. Section *Populus* in the study of Wu (1999) formed the second clade, and the taxa in this clade shared the same character of flat petiole in contrast to other taxa (Figure 6). The phylogenetic placements of the first and second clades were consistent with previously published phylogenetic relationships (Li et al., 2007; Wei et al., 2010). The results of Li et al. (2007) showed that section *Turanga* and section *Populus* can be divided into two independent groups based on AFLP markers, and the *trnL-F* sequence analysis also supported section *Populus* as a separate clade (Wei et al., 2010). In our morphology analysis, sections *Turanga and Populus* shared the same characters of bud size, but the characters of bark smoothness, petiole shape and leaf in inflorescence were different (Figure 6). Therefore, we can distinguish the two sections according to the three characters. Interestingly, our phylogenomic analysis showed that *P. nigra*, which was classified in section *Aigeiros* in the study of Wu (1999) is nested among the members of section *Populus*. The character of bud size different between *P. nigra* and *P. deltoides* (section *Aigeiros*), but the characters of small bud, flat petiole, no leaf in inflorescence, short anther length and cuneate anther tip were similar in section *Populus*. Therefore, the plastid data and morphology characteristics both revealed that *P. nigra* had a close affinity to species of section *Populus*, which supported the possibility of ancient hybridization by which *P. nigra* appeared to be an introgressant of the *P. alba* lineage and some other presently unknown paternal lineage of section *Populus* (Smith, 1988; Hamzeh and Dayanandan, 2004).

Section *Tacamahaca* in the study of Wu (1999) contained nearly 60 species and varieties that share the same characters of petiole eglandular, flower disk, and capsule usually glabrous or rarely pilose (Fang et al., 1999; Song, 2006). In our analysis, the members of section *Tacamahaca* were divided into clade III, clade IV and clade V, and the three clades shared the same character of a tetrete petiole. Clade III contained *P. haoana, P. kangdingensis, P. qamdoensis, P. schneideri, P. simonii, P. szechuanica, P. szechuanica* var. *tibetica, P. yunnanensis*, and the taxa of *P. lasiocarpa* and *P. pseudoglauca*, which had previously been retrieved as members of section *Leucoides* in the study of Wu (1999). Clade IV included the taxa *P. cathayana, P. koreana, P. laurifolia, P. trinervis, P. xiangchengensis, P. gonggaensis* and *P. wilsonii*, but the last two taxa were classified in section *Leucoides*. Clade V included *P. angustifolia, P. balsamifera, P. trichocarpa, P. deltoides, P. deltoides × nigra, P. fremontii*, and *P. mexicana*. All of the taxa in clade V had the character of a middle bud unlike the other two clades. Section *Aigeiros* in the study of Wu (1999), including *P. deltoides, P. deltoides* × *nigra*, and *P. fremontii*, was nested within clade V and shared the same characters of a middle bud, no leaf in inflorescence, short anther length and cuneate anther tip with the taxa of section *Tacamahaca* (Figure 6). Furthermore, the character of shallowly furrowed bark in *P. fremontii* was shared with *P. cathayana, P. koreana, P. laurifolia*, and *P. trinervis* which were members of section *Tacamahaca*. Therefore, our analysis did not support section *Aigeiros* as a sister section of sections *Populus, Tacamahaca* and *Turanga*. Curiously, *P. deltoides × nigra* was placed among the species of section *Aigeiros*, but the character of bud size was inherited from the parent *P. nigra* unlike the other members in clade V. Meanwhile, *P. mexicana* shared the four characters of petiole shape, leaf in inflorescence, male anther length and tip shape with section *Turanga*. Therefore, we speculated that *P. mexicana* may be caused by ancient hybridization or chloroplast capture from excluded samples or now-extinct North America poplar, similar to previous studies on two North America species *P. trichocarpa* and *P. balsamifera* (Huang et al., 2014; Liu et al., 2017).

Our analysis showed that clade V was a sister clade to clade IV. Clade V includes seven species (*P. angustifolia, P. balsamifera, P. deltoides, P. deltoides × nigra, P. fremontii, P. mexicana*, and *P. trichocarpa*) that are primarily distributed in North America. Clade IV comprises seven species that mainly exist within Eurasia. The phylogenetic relationships between clade IV and clade V matched well with the morphology data, such as bud size, although not well with smooth bark and petiole shape (Figure 6). The phylogenetic relationships between clade IV and clade V supported the hypothesis regarding the migration of species from Asia to North America (Tiffney and Manchester, 2001; Milne, 2006; Liu et al., 2017). The authors who proposed this hypothesis speculated that a common ancestor first appeared in North America and then dispersed to other continents via the North Atlantic Land Bridge and the Bering Land Bridge. Then, with the disappearance of the Bering Land Bridge, new species emerged due to the geographic isolation (Tiffney and Manchester, 2001; Milne, 2006; Liu et al., 2017).

The taxa in clade III and clade IV had an overlapping distribution, such as *P. xiangchengensis* and *P. gonggaensis* in clade IV, which were distributed in southwest China, while the taxa of *P. schneideri* and *P. kangdingensis* in clade III were also distributed in this area. Clade III and IV showed high similarity in morphology characters (Figure 6), thus complicating their distribution. Liu and Fu (2004) considered *P. xiangchengensis* a hybridization of *P. schneideri* and *P. pseudoglauca* based on morphological characteristics, while another study suggested that *P. xiangchengensis* was a likely hybrid species of *P. kangdingensis* and *P. pseudoglauca* (Wan et al., 2009). *P. schneideri* was also considered a natural hybrid formed by *P. kangdingensis* and *P. cathayana* (Chen et al., 2007; Wang, 2012). The uplift of the Qinghai-Tibet (Q-T) plateau began approximately 40 million years ago (Ma) following the collision of India and Asia (Chung et al., 1998). Some new niches were created by the uplifts, while other plants retreated in response to the climatic changes, thus providing strong opportunities for new hybrid species to emerge (Wang et al., 2005; Liu et al., 2006; Wang and Gilbert, 2007; Lu et al., 2014). Meanwhile, the uplift of the Q-T plateau also induced significant geographical effects (He et al., 2015). These complex factors account for section *Tacamahaca*’ s presence among the current phylogenetic relationships. Further research using more genetic data and more individuals per species is needed to deeply illuminate the complex relationships in section *Tacamahaca* in the future.

## Conclusion

This study clarified the phylogenetically intergenetic and interspecific relationships of *Populus*. The genus was confirmed as monophyletic and subdivided into five well-resolved and strongly supported clades. Clade I included three taxa: *P. euphratica, P. pruinosa* and *P. ilicifolia*. Clade II contained thirteen plastomes of the taxa *P. adenopoda, P. alba, P. bolleana, P. davidiana, P. hopeiensis, P. nigra, P. qiongdaoensis, P. rotundifolia, P. rotundifolia* var. *duclouxiana, P. tremula, P. tremula × alba, P. tomentosa* and *P. tomentosa* (NC). Clade III included ten taxa: *P. haoana, P. kangdingensis, P. lasiocarpa, P. pseudoglauca, P. qamdoensis, P. schneideri, P. simonii, P. szechuanica, P. szechuanica* var. *tibetica*, and *P. yunnanensis*. Clade IV included *P. cathayana, P. gonggaensis, P. koreana, P. laurifolia, P. trinervis, P. wilsonii* and *P. xiangchengensis*. The last clade was made up of *P. angustifolia, P. balsamifera, P. deltoides, P. deltoides × nigra, P. fremontii, P. mexicana*, and *P. trichocarpa*. Six morphological characters, including bark smoothness, petiole shape, bud size, leaf in inflorescence, and anther length and tip, agreed well with the molecular phylogenies and can be used to subdivide the *Populus* genus.

## Author Contributions

DZ performed the experiments, analyzed the data, wrote the manuscript, and prepared the figures and/or tables. PG performed the experiments and prepared the figures and/or tables. AZ, YZ, and XZ analyzed the data and reviewed drafts of the manuscript. AD, YS, and CH conceived and designed the experiments, reviewed the drafts of the manuscript, and approved the final draft. All authors read and approved the final manuscript.

## Conflict of Interest Statement

The authors declare that the research was conducted in the absence of any commercial or financial relationships that could be construed as a potential conflict of interest.
