# Supplementary material for: Plastome Sequences Help to Resolve Deep-Level Relationships of Populus in the Family Salicaceae
Source: Front Plant Sci. 2019 Jan 22;10:5. doi: 10.3389/fpls.2019.00005 (PMC6349946; doi:10.3389/fpls.2019.00005)
Supplement: Supplementary file 4 [file Data_Sheet_1.docx]

Supplementary Material

Plastome sequences help to resolve deep-level relationships of *Populus* in the family Salicaceae

Dan Zong^1,2^, Peihua Gan^1,2^, Anpei Zhou^1,2^, Yao Zhang^1,2^, Xinlian Zou^1,2^, Anan Duan^1,2,3^, Yu Song^4,5*^, Chengzhong He ^1,2,3*^

*** Correspondence:**

Yu Song: [songyu@xtbg.ac.cn](mailto:songyu@xtbg.ac.cn)

Chengzhong He: hecz@swfu.edu.cn

# Supplementary Tables

**Supplementary Table S1.** List of genes encoded by 24 *Populus* chloroplast genomes

**Supplementary Table S2.** Nucleotide variability values among the 40 *Populus* plastomes*.*

**Supplementary Table S3.** Pairwise nucleotide divergence among the 40 *Populus* plastomes*.*
